# Reliability of assessing skeletal muscle architecture and tissue organization of the gastrocnemius medialis and vastus lateralis muscle using ultrasound and spatial frequency analysis

**DOI:** 10.3389/fspor.2024.1282031

**Published:** 2024-01-18

**Authors:** Melanie Lesinski, Gregory Bashford, Adrian Markov, Lucie Risch, Michael Cassel

**Affiliations:** ^1^ Division of Training and Movement Sciences, Research Focus Cognition Sciences, University of Potsdam, Potsdam, Germany; ^2^ Department of Biological Systems Engineering, University of Nebraska, Lincoln, NE, United States; ^3^ Department of Sports Medicine, University Outpatient Clinic, University of Potsdam, Potsdam, Germany

**Keywords:** sonography, intraday intra-rater reliability, inter-rater reliability, interpretation error, muscle thickness, pennation angle, fascicle length

## Abstract

**Introduction:**

The purpose of this study was to investigate inter- and intra-rater reliability as well as the inter-rater interpretation error of ultrasound measurements assessing skeletal muscle architecture and tissue organization of the gastrocnemius medialis (GM) and vastus lateralis (VL) muscle.

**Methods:**

The GM and VL of 13 healthy adults (22 ± 3 years) were examined thrice with sagittal B-mode ultrasound: intraday test-retest examination by one investigator (intra-rater) and separate examinations by two investigators (inter-rater). Additionally, images from one investigator were analysed by two interpretators (interpretation error). Muscle architecture was assessed by muscle thickness [MT], fascicle length [FL], as well as superior and inferior pennation angle [PA]. Muscle tissue organization was determined by spatial frequency analysis (SFA: peak spatial frequency radius, peak −6 dB width, PSFR/P6, normalized peak value of amplitude spectrum [Amax], power within peak [PWP], peak power percent). Reliability of ultrasound examination and image interpretation are presented as intraclass correlation coefficient (ICC), test-retest variability, standard error of measurement as well as bias and limits of agreement.

**Results:**

GM and VL demonstrated excellent ICCs for inter- and intra-rater reliability, along with excellent ICCs for interpretation error of MT (0.91–0.99), showing minimal variability (<5%) and SEM% (<5%). Systematic bias for MT was less than 1 mm. For PA and FL poor to good ICCs for inter- and intra-rater reliability were revealed (0.41–0.90), with moderate variability (<12%), low SEM% (<10%) and systematic bias between 0.1–1.4°. Tissue organization analysis indicated moderate to good ICCs for inter- and intra-rater reliability. Notably, Amax and PWP consistently held the highest ICC values (0.77–0.87) across all analyses but with higher variability (<24%) and SEM% (<18%), compared to lower variability (<9%) and SEM% (<8%) in other tissue organization parameters. Interpretation error of all muscle tissue organization parameters showed excellent ICCs (0.96–0.999) with very low variability (≤1%) and SEM% (<2%), except Amax & PWP (TRV%: <6%; SEM%: <7%).

**Conclusion:**

Our findings demonstrated excellent inter- and intra-rater reliability for MT. However, agreement for PA, FL, and SFA parameters was not as strong. Additionally, MT and all SFA parameters exhibited excellent agreement for inter-rater interpretation error. Therefore, the SFA seems to offer the possibility of objectively and reliably evaluating ultrasound images.

## 1 Introduction

The architecture of human skeletal muscles plays an important role in its function (1–3). Studies have shown that muscle thickness (MT), fascicle length (FL) as well as fascicle pennation angle (PA) are important determinants for the force generating capacity of a particular muscle (1, 2, 4). Muscle architecture seems to be highly adaptable in response to different stimuli, which can partly explain the changes in function following training and injury (3). Significant alterations in muscle architecture are evident following both an acute bout of resistance exercise as well as long-term resistance training (3, 5–8). For instance, Vieira et al. (8) found that the fatigue-induced drop in performance due to an acute bout of concentric isokinetic knee extension exercises were associated with changes in muscle architecture (VL: MT +11%–14%, PA: +39%). Limited evidence exists to characterise the effect of injury on muscle architecture. Timmins et al. (9) provided evidence for shorter fascicles and greater pennation angles in individuals with a history of strain injury. Due to a lack of prospective studies, it is still unclear whether these architectural changes are the cause or consequence of injury.

To investigate human skeletal muscle architecture (i.e., MT, FL, and PA) *in vivo*, B-mode ultrasound imaging has become a popular method due to its inexpensive, portable, safe, and non-invasive nature (10). Recently, spatial frequency analysis (SFA) of ultrasound images has been also used to assess micromorphological characteristics such as tissue density and organization of tendons resulting from pathological or training related adaptations (11, 12). Due to similar hierarchical structure of tendon and muscle tissue, a recent investigation successfully adapted and extended SFA for the application in skeletal muscle (13, 14). In a first examination Crawford et al. (13) accomplished a characterization of muscle injury and recovery due to SFA parameters by providing quantitative information on both fascicular disruption and edema presence in acute hamstring strain injury. Afterwards, Crawford et al. (15) conducted a reliability study investigating hamstring muscles and focusing on four SFA parameters (i.e., PSFR, Mmax, Mmax%, Sum). They found excellent intraclass correlation coefficients (ICCs) for the inter-rater interpretation error between different interpreters for the extracted spatial frequency parameters (ICC: 0.95–0.98). Therefore, SFA may be an objective method to determine training induced acute and chronic micromorphological adaptations or changes in skeletal muscles organization due to injury and pathologies.

Methodological limitations of ultrasound imaging include inconsistency of image acquisition [probe placement, probe rotation, probe orientation (e.g., angle), and probe pressure] and interpretation. Whether or not a standardization protocol is sufficient to overcome these methodological limitations can be assessed by the investigation of inter-rater reliability (investigator 1 vs. investigator 2), intra-rater reliability (measurement 1 vs. measurement 2 of one investigator) as well as interpretation error (same image: interpretator 1 vs. interpretator 2).

Ten years ago Kwah et al. (16) conducted a systematic review on the reliability and validity of ultrasound measurements of PA and FL in human muscles. Among other subanalyses, they took a closer look on the inter- and intra-rater reliability as well as the inter-rater interpretation error. They found good to excellent inter-rater reliability (PA: ICC 0.80; FL: ICC 0.80–0.97), moderate to excellent intra-rater reliability (PA: ICC 0.51–1.00; FL: ICC 0.62–0.99) as well as good to excellent interpretation error (PA: 0.85–1.00; FL: ICC 0.87–0.99) of the measurement of PA and FL in humans. However, Kwah et al. (16) summarized findings from studies that investigated different muscles (e.g., shoulder, arm, shank, and thigh muscles) and populations (e.g., live subjects vs. cadavers). The feasibility of the ultrasound measurement is muscle-specific due to different muscle localization and size. Therefore, it is assumed that the reliability of ultrasound measurement is also muscle-specific. The gastrocnemius medialis (GM) and vastus laterlis (VL) are two of the main locomotor muscles and active for instance during walking, running, and jumping. Particularly for the GM and VL muscle, no study exists that comprehensively investigates the inter- and intra-rater reliability as well as the inter-rater interpretation error of ultrasound measurement to assess the muscle architecture (i.e., MT, FL, PA) and especially muscle tissue organization using SFA of these two muscles. Furthermore, the assessment of measurement errors in our laboratory setup is vital for accurately interpreting potential intervention effects in subsequent intervention studies.

Hence, the aim of this study was to assess the inter- and intra-rater reliability as well as inter-rater interpretation error of ultrasound skeletal muscle architecture and tissue organization measurements using ultrasound and SFA of the GM and VL muscle in healthy young adults.

## 2 Material and methods

### 2.1 Study design

**Figure 1:**
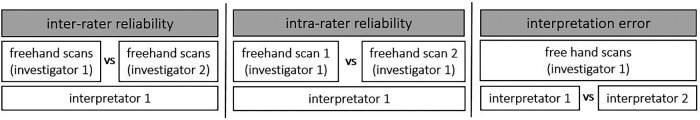
A single-group study design was conducted to examine inter-rater and intraday intra-rater reliability as well as inter-rater interpretation error of the skeletal muscle architecture and tissue organization assessment via B-mode ultrasound scans.

A single-group study design was conducted to examine inter-rater and intraday intra-rater reliability as well as inter-rater interpretation error of sonographic examinations evaluating skeletal muscle architecture and tissue organization. To determine inter-rater reliability of ultrasound assessment, all participants were examined by two investigators independently (investigator 1 vs. investigator 2; Figure 1). Both investigators held the probe manually at the predefined location (for more details see below). Both Investigators have several weeks/month of experience with sonographic assessments. According to König et al. (17), investigators with only several weeks/month of experience can assess muscle architecture with good to high reliability. To allow intraday intra-rater reliability assessment, all participants were measured twice by the same investigator (investigator 1: measurement 1 vs. measurement 2; Figure 1). To assess inter-rater interpretation error, ultrasound scans were interpreted twice (same image: interpretator 1 vs. interpretator 2; Figure 1).

### 2.2 Participants

Thirteen healthy young, physically active adults (i.e., sport students) aged 19 to 30 years volunteered to participate in this study (Table 1). Exclusion criteria were defined *a priori* as any musculoskeletal, neurological, and/or orthopedic disorders in the lower extremities that occurred within the last six months prior to the start of the study. Written informed consent was obtained from all participants before study inclusion. The study was approved by the local ethics committee. All experiments were conducted according to the latest version of the declaration of Helsinki (18).

**Table 1:** Characteristics of study participants.

	particpants (all *N* = 13)
Gender	7 males/6 females
Age (years)	22 ± 3 (range: 19–30)
Body height (cm)	174 ± 8 (range: 161–184)
Body weight (kg)	69.7 ± 10.1 (range: 54.9–83.3)
Muscle mass (kg)	33.4 ± 6.9 (range: 23.3–44.5)
Fat mass (%)	15.7 ± 5.5 (range: 6.3–23.4)
Body mass index (kg/m²)	23.0 ± 1.9 (range: 19.2–25.9)

### 2.3 Measurement procedure

**Figure 2:**
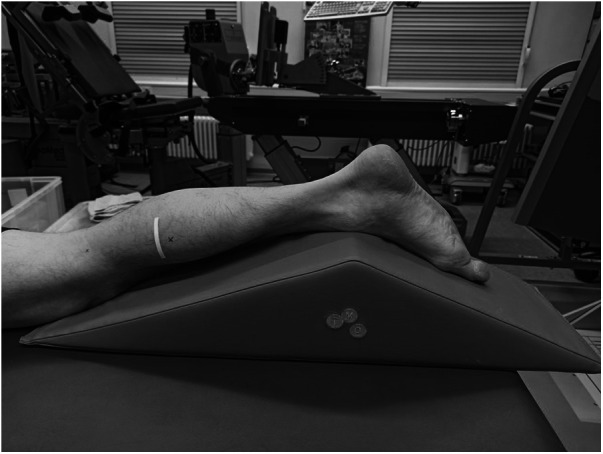
Measurement location of the right gastrocnemius medialis muscle.

**Figure 3:**
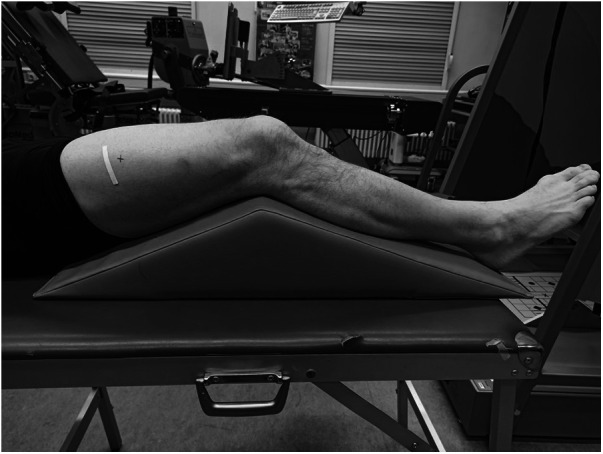
Measurement location of the right vastus laterlis muscle.

At the beginning of the testing session, anthropometric and body composition tests were performed under strictly standardized conditions. Body composition was analyzed with the InBody720 (Biospace; Seoul, South Korea). Afterwards the participants laid prone on an examination table with the right leg supported on an inclined foam wedge (ankle 40° of plantar flexion; Figure 2) to assess the right GM muscle. For assessing the right VL muscle, participants laid supine on an examination table with the right leg supported on an inclined foam wedge (knee 25° flexed; Figure 3) to avoid tension in the VL muscle. The knee and ankle joint angles were consistent within each subject during repeated measurements because the wedge was consistently placed in the same position. GM and VL were assessed under resting condition. Longitudinal ultrasound scans (Vivid q; GE Healthcare, Tirat Carmel, Israel) of the GM and VL muscle belly were conducted with a 7.5-MHz linear ultrasound array (6 cm, 4–13 MHz). The preset was standardized (frequency: 11 MHz; depth: 4.5 cm; gain: 38%; dynamic range: 102; foci for GM: 1.2 cm and 2.5 cm, foci for VL: 1.625 cm and 3.125 cm) and kept constant for all image acquisitions. Care was taken to apply minimal pressure on the probe to prevent compression of the muscle.

To standardize the measurement location of the muscles, the placement of the probe was marked for GM and VL using a marker pen. The GM location was defined at a point one-third of the distance between the popliteal crease (tendon of semitendinosus muscle) and the medial malleolus. The VL location was defined half-way between the mid of patella and the greater trochanter (19). To recover the marked location in the ultrasound images, a thin strip of echoabsorptive tape was placed 1.5 cm proximal above the previously marked location (20). The probe was aligned longitudinally to the leg.

**Figure 4:**
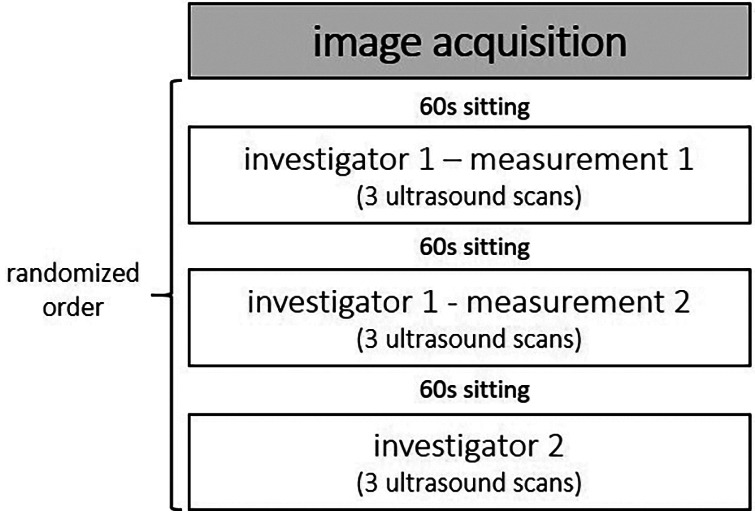
Image acquisition conditions.

After image acquisitions were completed from one investigator, the participant sat on the exam table for 60 s (15), before laying back down to be measured again by the same investigator (measurement 2) or the second investigator, respectively. The measurements were performed in randomized order (Figure 4). For each condition, 3 scans of the GM and VL muscle were conducted at the same location, resulting in a total of 117 images for each muscle.

### 2.4 Data analysis

**Figure 5:**
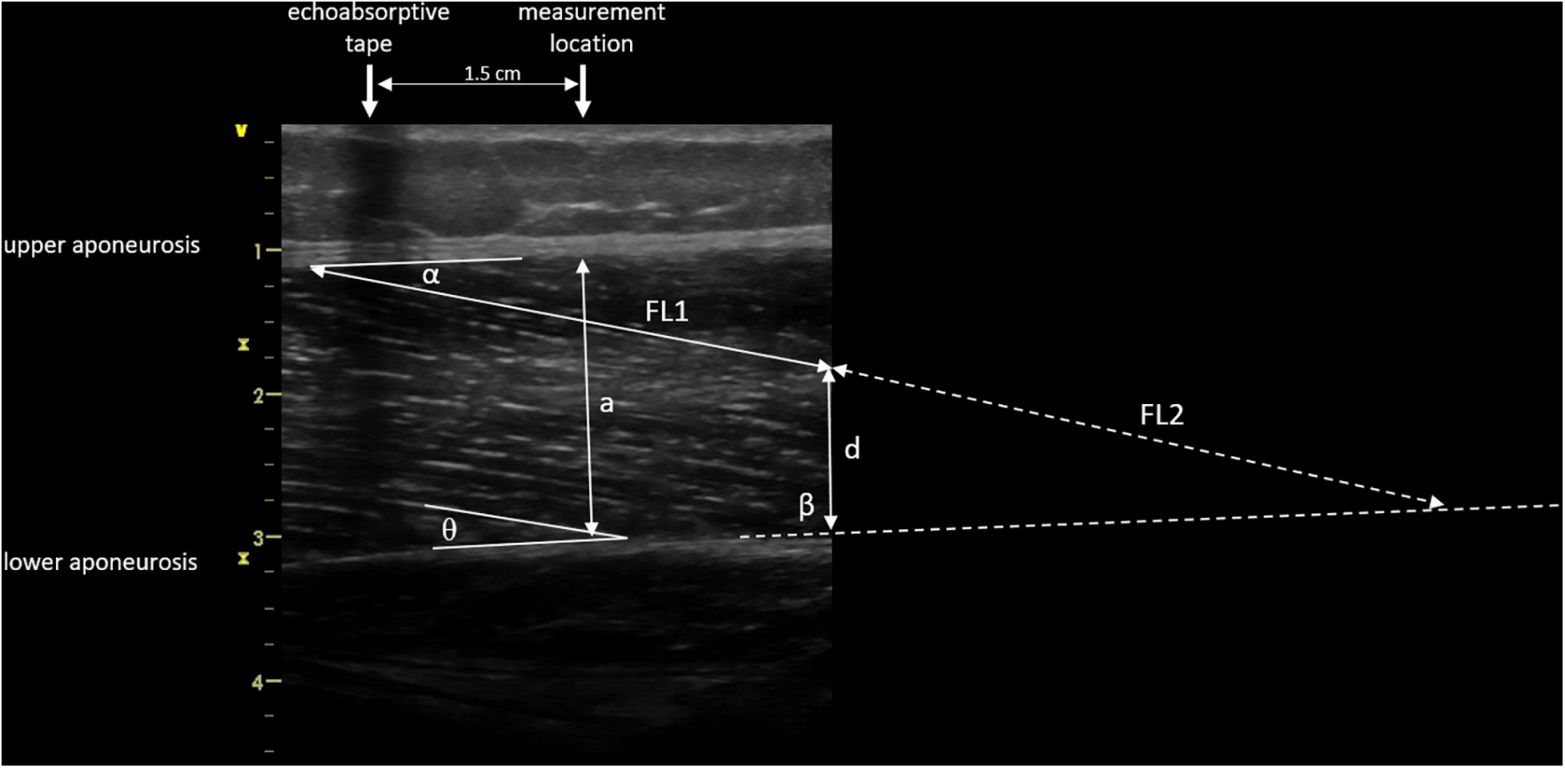
Muscle architecture parameters: muscle thickness 1.5 cm distal from echoabsorptive tape (**a**), fascicle length (visible length (FL1) + calculated length (FL2) as described by Baudry et al. (21)), superior pennation angle (α), inferior pennation angle (*θ*), angle between the inferior aponeurosis and the image boarder (β), and height between the inferior aponeurosis and the most distal part of the fascicle in the field of view (**d**).

Images were saved and transferred to a computer. Free Java-based ImageJ software (National Institutes of Health, Bethesda, MD, Version:1.53 s) was used to analyze the skeletal muscle architecture (i.e., MT, inferior/superior PA, FL; Figure 5). Due to a calibration image, the Image J software was provided with precise information regarding the specific distance in the captured images that corresponds to 1 cm. After this calibration of the system, distances and angles can be determined by simply clicking on points in the image. Superior PA was measured as the angle between the upper aponeurosis and the fascicle. Inferior PA was measured as the angle between the lower aponeurosis and the fascicle. MT was measured at the predefined position (1.5 cm distal from the echoabsorptive tape) by taking the distance between the upper and lower aponeuroses.

In case the fascicle extended the field of view during the ultrasound, according to Baudry et al. (21) FL is calculated from the visible FL (FL1) plus the calculated FL (FL2):

$$\mathrm{FL}2=\frac{d*\sin\beta }{\sin\theta }$$

where d is the height between the inferior aponeurosis and the most distal part of the fascicle in the field of view, *β* is the angle between the inferior aponeurosis and the image border, and *θ* is the inferior PA.

To analyze tissue organization, all ultrasound images were imported into MATLAB (Mathworks, USA R2016a) to conduct the SFA described by Bashford et al. (11). The SFA is a quantitative ultrasound method, which analyzes the anisotropic B-mode speckle pattern arising from within a tissue type in the spatial frequency domain. Briefly, a polygonal region of interest (ROI) is manually drawn in an image, and within this ROI, smaller sub-regions termed “kernels” are analyzed in the spatial frequency domain. For each kernel that fits within the ROI, parameters extracted from the FFT-derived spatial frequency spectral estimate are extracted. The parameters may be used for statistical comparisons such as classification. This method was recently updated by Bashford (unpublished work, 2023). In the previous method, the maximum value within each spatial spectrum was used to identify a spectral region of interest. In the new method, each spectral estimate *F*(*u*,*v*) is modelled as a two-dimensional narrowband spatial signal with a dominant spatial peak consisting of an elliptical Gaussian:

$$F(u,v)=A_{m}(-(a{\left(u-u_{m}\right)}^{2}+2b(u-u_{m})(v-v_{m})+c{\left(v-v_{m}\right)}^{2}))$$

**Figure 6:**
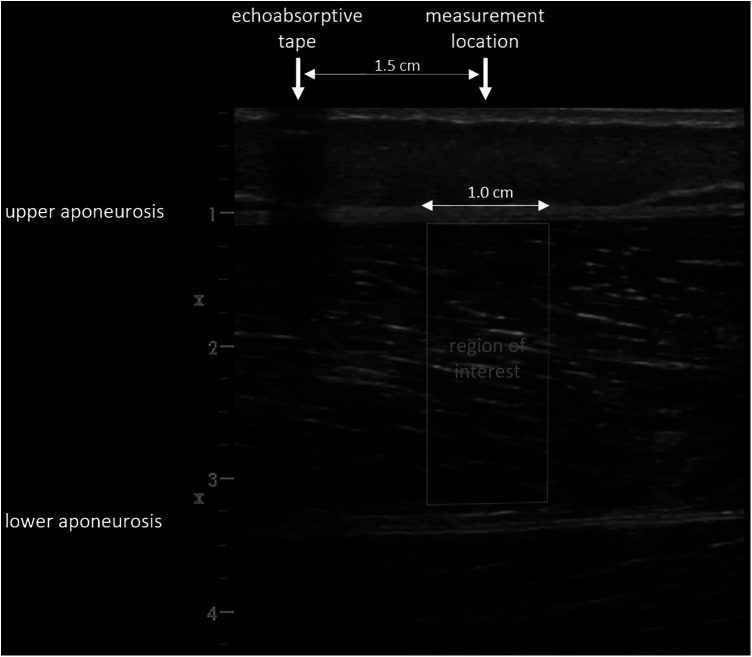
Selected region of interest for the spatial frequency analysis.

Where *A_m_* denotes the amplitude of the elliptical Gaussian, (*u_m_*,*v_m_*) denotes the center of the elliptical Gaussian, and helper variables *a*, *b*, and *c*

$$\begin{array}{rl} & a=\frac{\cos^{2}\theta }{2\sigma_{u}^{2}}+\frac{\sin^{2}\theta }{2\sigma_{v}^{2}}\\ & b=-\frac{\sin\;2\theta }{4\sigma_{u}^{2}}+\frac{\sin\;2\theta }{4\sigma_{v}^{2}}\\ & c=\frac{\sin^{2}\theta }{2\sigma_{u}^{2}}+\frac{\cos^{2}\theta }{2\sigma_{v}^{2}} \end{array}$$

describe the spread; here ($\sigma_{u}^{2},\;\sigma_{v}^{2}$) are the variances of the Gaussian in the *u*- and *v*- directions respectively, and *θ* is the rotation of the Gaussian ellipsoid. The spectral data was fit to the ellipsoidal Gaussian using an unconstrained multivariable simplex method (22). Spatial frequency parameters, including four new parameters (Table 2), were extracted from the model after fitting was achieved. The kernel dimensions (number of pixels) was set the same as Bashford et al. (11) as the structures under analysis were at similar image depths and similar ROI sizes. A standardized ROI was selected by measuring a 1 cm wide rectangular area of the GM and VL from the upper to the lower aponeuroses at the muscle belly, at a predefined position located such that the center of the ROI was 1.5 cm from the echoabsorptive tape; i.e., the vertical edges of the ROI were 1 cm and 2 cm distal from the echoabsorptive tape (Figure 6). The SFA analysis yielded six spatial frequency parameters: peak spatial frequency radius [PSFR], peak −6 dB width [P6], PSFR/P6 [Q6], normalized peak value of amplitude spectrum [Amax], power within peak [PWP], and peak power percent [PPP] (Table 2).

**Table 2:** Mathematical description and physiological correlate of spatial frequency parameters.

Parameter	Mathematical description	Mathematical formulation	Physiological correlates
Peak Spatial Frequency Radius (PSFR)	Distance from origin to peak of maximum frequency amplitude in 2-D FFT spectrum	$\sqrt{u_{m}^{2}+v_{m}^{2}}$, where $\left(u_{m},v_{m}\right)$ is the location of the model spectral peak	• Most dominant spacing between hyperechoic perimysium of the muscle fascicles and hypoechoic muscle fibers • Higher value primarily indicates a tighter packing of tendon/muscle fibers
P6 Width	Euclidian distance of the standard deviation vector of the spatial frequency peak on 2-D FFT spectrum	$\sqrt{\sigma_{u}^{2}+\sigma_{v}^{2}}$, from model fit	• A higher P6 value indicates more disorganization of tendon/muscle fibers
Q6 Factor	Ratio of Peak Spatial Frequency Radius to P6 Width	Q6 = $\frac{\mathrm{PSFR}}{P6}$	• Normalization factor to faciliate comparision of fiber packing with fiber alignment • Higher value primarily indicates a “purer” (parallel) alignment of tendon/muscle fibers.
Amax	Normalized peak value of amplitude spectrum	$\frac{F_{\max}(u,v)}{numel(F)}$	• Strength factor of the most dominant spacing of fascicles/fibers • Higher value primarily indicates more tendon/muscle fibers per unit volume
PWP	Image power within dominant spacing peak	$\int_{S}\frac{{\left|F(u,v)\right|}^{2}}{numel(F)}$, where *S* is the region of the dominant narrowband peak	• Strength factor of fiber spacing close to most dominant spacing • Higher value primarily indicates more tendon/muscle fibers per unit volume
PPP	Peak power percent	$\frac{\mathrm{PWP}}{2\int {\left|F(u,v)\right|}^{2}}\times 100\text{\%}$	• Strength factor of most dominant spacing of fascicles/fibers as compared to other tissue • Higher value primarily indicates more tissue in alignment compared to other tissue in the sample volume

To ensure blinded image evaluation, all images were coded with randomly assigned numbers throughout the entire interpretation process using a software that additionally reordered the images. The analysis was consequently carried out in a shuffled sequence without any knowledge of the participant ID, the investigator, the number of measurements, or trial. Following the image analysis, the measured results were then matched to the participants based on the random number codes in the master file.

### 2.5 Statistical analysis

Patient characteristics as well as muscle outcome parameters are presented descriptively by mean ± standard deviation (SD). For muscle parameters, the respective means of the 3 scans for each condition (Figure 4) were calculated and used for further statistical analysis. To assess inter-rater and intraday intra-rater comparisons as well as interpretation error, the ICC 2.1 (23) was calculated, defining the level of reliability as poor (ICC < 0.5), moderate (0.5 ≤ ICC < 0.75), good (0.75 ≤ ICC ≤ 0.9), or excellent (ICC > 0.9) (23). Furthermore, Bland-Altman analyses were conducted to determine the Bias (mean difference) and the 95% limits of agreement (LoA). To ensure valid LoA data, homoscedasticity was checked by applying Pearson Moment Correlation with the variables “mean” and “difference” of the two measurements (24). If homoscedasticity was not present in a data set, LoA derived from log transformed data that were finally back-transformed to give LoA for the ratio of the actual measurements (24). Moreover, test-retest variability (TRV%) was calculated by $\left|\frac{\mathrm{difference}}{\mathrm{mean}}\right|\times \;100\text{\%}$ for each participant. The level of variability was defined as very low (TRV% < 5%), low (5% ≤ TRV% < 10%), moderate (10% ≤ TRV% < 20%), and high (TRV% > 20%). To provide an estimate of the precision of measurements that keeps the unit of the parameter, standard error of measurement (SEM) was calculated as follows: $\mathrm{SD}\;\times \;\sqrt{\left(1-r\right)}$; where SD is the average standard deviation of the means of the 2 measurements and r is the calculated ICC (25, 26). SEM% was calculated by $\frac{\mathrm{SEM}\;\times \;100\text{\%}}{\mathrm{mean}}$; where mean is the mean score of all trials. The calculation of the ICCs, Pearson moment correlations, and log transformations was performed with IBM SPSS Statistics Version 26.0 (IBM Corporation, Armonk, NY). The calculation of Bias, LoA, TRV%, SEM, and SEM% was performed in Microsoft Excel 2016 (Microsoft Corporation, Redmond, WA).

## 3 Results

### 3.1 Muscle architecture and tissue organization parameters and its inter-rater and intraday intra-rater reliability

The mean values ± SD, as well as the calculated parameters of inter- and intra-rater reliability for GM and VL muscle architecture and tissue organization, are presented in Tables 3, 4 as well as in the Supplementary Files (Supplementary Figures S1–S4). The analyses indicated excellent ICCs (0.91–0.99) for inter- and intra-rater reliability of MT for both muscles, with very low variability (TRV%: <5%), a systematic bias of −0.2 to 0.7 mm as well as SEM% of 1.5 to 4.2%. Furthermore, the analyses revealed poor to good ICCs (0.41–0.79) for inter- and intra-rater reliability of PA and FL for GM, with low variability (TRV%: <9%) and SEM% (<7%). Systematic bias was between 0 and 1° for PA and between 1.2 to 1.5 mm for FL. For VL, good ICC values (0.82–0.90) were obtained for inter- and intra-rater reliability of PA and FL, with moderate variability (TRV%: <12%) and low SEM% (<10%). Systematic bias was in maximum 0.4° for PA and between 0.3 to 3.3 mm for FL. Regarding GM and VL muscle tissue organization parameters, analyses showed moderate to good ICCs for inter- and intra-rater reliability (ICCs: 0.58–0.87), except for PPP of GM for inter-rater reliability (ICC: 0.29) and P6 of VL for intra-rater reliability (ICC: 0.37). Amax and PWP consistently showed the highest ICC values (0.77–0.87) across all analyses but also high variability (TRV%: <24%) and SEM% (7.4%–17.4%) compared to low variability (TRV%: <9%) and SEM% (<8%) of all other tissue organization parameters. For systematic bias and LoA of SFA parameters see Tables 3, 4.

**Table 3:** Gastrocnemius medialis and vastus lateralis muscle architecture and tissue organization and its inter-rater reliability (investiagtor 1 vs. investigator 2; interpretator 1).

	Parameter	Investigator 1	Investigator 2	ICC	TRV, %	Bias ± LoA	SEM	SEM, %
Gastrocnemius medialis	Muscle thickness [cm]	2.12 ± 0.25	2.19 ± 0.27	0.99	3.5	0.07 ± 0.12	0.03	1.5
	Superior pennation angle [°]	28.3 ± 2.5	28.3 ± 2.4	0.49	6.8	0.04 ± 4.9	1.5	5.3
	Inferior pennation angle [°]	30.0 ± 2.7	29.6 ± 2.9	0.72	6.1	−0.4 ± 4.2	1.4	4.6
	Fascicle length [cm]	4.50 ± 0.44	4.66 ± 0.40	0.79	5.5	0.15 ± 0.48	0.18	4.0
	PSFR [mm^−1^]	0.67 ± 0.07	0.66 ± 0.06	0.77	8.7	−0.01 ± 0.15	0.02	3.6
	P6 [mm^−1^]	0.88 ± 0.04	0.88 ± 0.05	0.68	3.4	0.00 ± 0.08	0.02	2.7
	Q6	0.94 ± 0.10	0.92 ± 0.12	0.58	9.3	0.02 ± 0.20	0.06	6.7
	Amax [B/sample]	0.70 ± 0.13	0.68 ± 0.16	0.84	8.8	0.02 ± 0.16	0.06	8.1
	PWP [B²]	3,350 ± 945	3,187 ± 1296	0.79	16.8	163 ± 1453	490	15.0
	PPP [%]	69.3 ± 3.5	69.3 ± 2.9	0.29	4.5	−0.01 ± 7.5	2.1	3.1
Vastus lateralis	Muscle thickness [cm]	2.41 ± 0.35	2.48 ± 0.34	0.91	4.4	0.07 ± 0.27	0.10	4.2
	Superior pennation angle [°]	13.1 ± 3.6	13.0 ± 3.8	0.87	10.9	−0.1 ± 3.8	1.3	9.9
	Inferior pennation angle [°]	13.5 ± 2.6	13.1 ± 2.3	0.82	9.4	−0.4 ± 2.8	1.0	7.3
	Fascicle length [cm]	11.14 ± 2.54	11.11 ± 2.95	0.84	11.2	−0.03 ± 3.15	1.05	9.5
	PSFR [mm^−1^]	0.91 ± 0.13	0.89 ± 0.12	0.61	9.2	−0.02 ± 0.21	0.07	7.5
	P6 [mm^−1^]	0.82 ± 0.04	0.83 ± 0.04	0.68	3.3	−0.01 ± 0.06	0.02	2.5
	Q6	1.30 ± 0.15	1.27 ± 0.15	0.63	7.3	0.03 ± 0.26	0.08	6.4
	Amax [B/sample]	0.77 ± 0.17	0.70 ± 0.18	0.77	13.7	0.06 ± 0.21	0.08	10.9
	PWP [B²]	3,903 ± 1333	3,409 ± 1473	0.77	23.9	495 ± 1707	638	17.4
	PPP [%]	64.9 ± 3.6	64.6 ± 5.3	0.68	4.4	0.3 ± 7.9	2.3	3.5

**Table 4:** Gastrocnemius medialis and vastus lateralis muscle architecture and tissue organization and its intraday intra-rater reliability (investigator 1: measurement 1 vs. measurement 2; interpretator 1).

	Parameter	Measurement 1	Measurement 2	ICC	TRV, %	Bias ± LoA	SEM	SEM, %
Gastrocnemius medialis	Muscle thickness [cm]	2.12 ± 0.25	2.10 ± 0.26	0.97	2.7	−0.02 ± 0.13	0.04	2.1
	Superior pennation angle [°]	28.3 ± 2.5	27.3 ± 3.2	0.49	7.6	−1.0 ± 5.7	1.8	6.4
	Inferior pennation angle [°]	30.0 ± 2.7	28.6 ± 3.0	0.41	8.7	−1.4 ± 5.9	1.9	6.4
	Fascicle length [cm]	4.50 ± 0.44	4.63 ± 0.38	0.58	6.5	0.12 ± 0.73	0.24	5.2
	PSFR [mm^−1^]	0.67 ± 0.07	0.69 ± 0.06	0.63	6.8	−0.02 ± 0.10	0.03	4.9
	P6 [mm^−1^]	0.88 ± 0.04	0.87 ± 0.04	0.79	2.6	0.01 ± 0.06	0.02	2.1
	Q6	0.94 ± 0.10	0.98 ± 0.09	0.72	6.9	−0.04 ± 0.12	0.05	4.8
	Amax [B/sample]	0.70 ± 0.13	0.72 ± 0.15	0.82	9.6	−0.02 ± 0.2	0.1	7.9
	PWP [B²]	3,350 ± 945	3,567 ± 1126	0.78	17.3	−217 ± 1341	461	13.3
	PPP [%]	69.3 ± 3.5	69.5 ± 3.5	0.65	3.3	−0.2 ± 5.8	1.9	2.7
Vastus lateralis	Muscle thickness [cm]	2.41 ± 0.35	2.40 ± 0.35	0.93	4.3	−0.01 ± 0.26	0.09	3.8
	Superior pennation angle [°]	13.1 ± 3.6	12.7 ± 3.0	0.90	9.8	−0.4 ± 2.9	1.0	7.8
	Inferior pennation angle [°]	13.5 ± 2.6	13.3 ± 2.3	0.82	9.8	−0.3 ± 3.0	1.0	7.4
	Fascicle length [cm]	11.14 ± 2.54	10.81 ± 2.20	0.84	8.2	−0.33 ± 2.64	0.91	8.3
	PSFR [mm^−1^]	0.91 ± 0.13	0.86 ± 0.11	0.66	7.5	−0.05 ± 0.18	0.06	7.1
	P6 [mm^−1^]	0.82 ± 0.04	0.80 ± 0.03	0.37	4.1	0.01 ± 0.08	0.02	2.8
	Q6	1.30 ± 0.15	1.28 ± 0.13	0.68	6.7	0.02 ± 0.23	0.07	5.6
	Amax [B/sample]	0.77 ± 0.17	0.81 ± 0.17	0.87	9.2	−0.04 ± 0.16	0.06	7.4
	PWP [B²]	3,903 ± 1333	4,151 ± 1495	0.86	14.9	−247 ± 1441	510	12.7
	PPP [%]	64.9 ± 3.6	66.3 ± 4.1	0.77	3.0	−1.4 ± 4.7	1.8	2.7

### 3.2 Muscle architecture and tissue organization parameters and its inter-rater interpretation error

The mean values ± SD as well as the calculated parameters of inter-rater interpretation error for GM and VL muscle architecture and tissue organization, are presented in Table 5 and in the Supplementary Files (Supplementary Figures S1–S4). The analyses indicated excellent ICCs (0.96–0.99) for inter-rater interpretation error for MT and all tissue organization parameters for both muscles. Variability (TRV%: <1%) and SEM% (<2%) for these parameters were very low, except for Amax and PWP (TRV%: <6%; SEM%: <7%). Systematic bias for MT was in maximum 0.1 mm. For SFA parameter-specific systematic bias and LoA see Tables 5. Regarding PA and FL both muscles analyses showed moderate to good ICCs (0.69–0.87) for the inter-rater interpretation error with moderate variability (TRV%: <12%) and low SEM% (<10%), except excellent ICC (0.94) for superior PA of VL. Systematic bias of PA were less than 1° and between 3.2–6.5 mm for FL.

**Table 5:** Gastrocnemius medialis and vastus laterlis muscle architecture and tissue organization and its inter-rater interpretation error (interpretator 1 vs. interpretator 2).

	Parameter	Interpretator 1	Interpretator 2	ICC	TRV, %	Bias ± LoA	SEM	SEM, %
Gastrocnemius medialis	Muscle thickness [cm]	2.12 ± 0.25	2.12 ± 0.25	0.999	0.4	0.00 ± 0.02	0.01	0.4
	Superior pennation angle [°]	28.3 ± 2.5	29.2 ± 3.4	0.87	4.9	−0.9 ± 2.7	1.1	3.7
	Inferior pennation angle [°]	30.0 ± 2.7	30.8 ± 3.3	0.75	6.9	−0.8 ± 4.8	1.7	5.4
	Fascicle length [cm]	4.50 ± 0.44	4.18 ± 0.42	0.69	7.8	0.32 ± 0.43	0.23	5.4
	PSFR [mm^−1^]	0.67 ± 0.07	0.67 ± 0.06	0.99	0.9	0.00 ± 0.02	0.01	0.9
	P6 [mm^−1^]	0.88 ± 0.04	0.88 ± 0.04	0.99	0.3	0.00 ± 0.01	0.00	0.4
	Q6	0.94 ± 0.10	0.94 ± 0.09	0.99	1.0	0.00 ± 0.03	0.01	1.2
	Amax [B/sample]	0.7 ± 0.1	0.7 ± 0.1	0.998	0.8	0.00 ± 0.02	0.01	0.8
	PWP [B²]	3,350 ± 945	3,334 ± 935	0.997	1.6	16 ± 137	51	1.5
	PPP [%]	69.3 ± 3.5	69.3 ± 3.2	0.99	0.4	−0.01 ± 0.9	0.3	0.4
Vastus lateralis	Muscle thickness [cm]	2.41 ± 0.35	2.42 ± 0.36	0.997	0.6	−0.01 ± 0.05	0.02	0.8
	Superior pennation angle [°]	13.1 ± 3.6	13.4 ± 3.5	0.94	7.7	−0.3 ± 2.4	0.8	6.4
	Inferior pennation angle [°]	13.5 ± 2.6	14.2 ± 3.0	0.76	11.9	−0.6 ± 3.8	1.3	9.4
	Fascicle length [cm]	11.14 ± 2.54	10.19 ± 2.29	0.85	10.5	−0.65 ± 2.27	0.87	8.0
	PSFR [mm^−1^]	0.91 ± 0.13	0.91 ± 0.13	0.998	0.8	0.00 ± 0.02	0.01	0.6
	P6 [mm^−1^]	0.82 ± 0.04	0.81 ± 0.04	0.988	0.7	0.01 ± 0.01	0.00	0.2
	Q6	1.30 ± 0.15	1.31 ± 0.16	0.99	1.0	−0.01 ± 0.04	0.02	1.3
	Amax [B/sample]	0.77 ± 0.17	0.76 ± 0.18	0.98	2.5	−0.01 ± 0.07	0.02	3.0
	PWP [B²]	3,903 ± 1333	3,845 ± 1534	0.97	5.4	−66 ± 757	265	6.8
	PPP [%]	64.9 ± 3.6	64.7 ± 3.7	0.96	0.9	−0.3 ± 2.0	0.7	1.1

## 4 Discussion

The aim of this study was to assess inter-rater and intraday intra-rater reliability as well as inter-rater interpretation error of ultrasound skeletal muscle architecture and tissue organization measurements using ultrasound and SFA of GM and VL muscle in healthy young adults. Findings revealed that GM had lower MT, shorter FL as well as greater PA compared to VL. In terms of reliability testing, GM and VL showed excellent ICC values for inter-rater and intraday intra-rater reliability as well as excellent ICCs for inter-rater interpretation error for MT, with very low variability and SEM% (<5%). Systematic bias for MT was less than 1 mm. Furthermore, the analyses revealed poor to good ICCs for inter-rater and intraday intra-rater reliability of PA and FL for both muscles, with moderate variability (<12%), low SEM% (<10%) and systematic bias between 0.1 to 1.4°. Reliability testing of tissue organization indicated moderate to good ICCs for inter-rater and intraday intra-rater reliability. Amax and PWP consistently showed the highest ICC values (0.77–0.87) across all analyses, but also the highest variability (<24%) and SEM% (<18%), compared to the low variability (<9%) and SEM% (<8%) of all other tissue organization parameters. In particular for the inter-rater interpretation error, ICCs of all muscle tissue organization parameters were excellent with very low variability (≤1%) and SEM% (<2%) between interpretators, except Amax and PWP (TRV%: <6%, SEM%: <7%).

Several years ago, Kwah et al. (16) conducted a systematic review indicating good to excellent ICCs regarding the inter-rater reliability, moderate to excellent ICCs for the intra-rater reliability as well as good to excellent ICCs concerning interpretation error of ultrasound measurements of muscle architecture (i.e., PA and FL) in humans. However, they summarized findings from studies that investigated different muscles (e.g., shoulder, arm, shank, and thigh muscles), populations (e.g., *in vivo* vs. cadaver), and basis of calculation (e.g., single scan vs. mean of 3 scans). To have a closer look, in particular, on inter- and intra-rater reliability as well as inter-rater interpretation error of GM and VL ultrasound imaging in live humans, there exist only few studies (17, 27–30). For instance, König et al. (17) investigated inter-rater reliability as well as inter-rater interpretation error of ultrasound measurements (in clinical settings) of the GM architecture in healthy female and male adults (6 males: 29 ± 5 years, 9 females: 28 ± 3 years). They found good inter-rater reliability (ICC: 0.77–0.90; SEM: 0.1 cm [MT], 1.0–1.1° [PA], 0.4 cm [FL]) and good to excellent interpretation error (ICC: 0.76–0.96; SEM: 0.05 cm [MT], 1.3–1.7° [PA], 0.2 cm [FL]). They used the mean of 3 scans of each participants for their analysis and their findings are comparable to the present results for GM. Concerning intra-rater reliability, Raj et al. (29) investigated the ultrasound measurements of GM and VL muscle architecture in older adults (11 males, 10 females; 68 ± 5 years) and used the mean of 3 scans of each participants for their analysis. Measures were taken on two separate occasions and indicated good to excellent ICC values (0.80–0.97) in terms of intra-rater test-retest reliability for MT, PA, and FL of GM and VL muscle. Likewise, May et al. (27) investigated the intra-rater test-retest reliability of ultrasonographic measurement of GM muscle architecture. They examined 87 participants (44 males, 43 females; 22 ± 9 years) on two separate occasions and found moderate to excellent ICCs (0.63–0.91) regarding the intra-rater test-retest reliability for MT, PA, and FL. These findings are comparable to the present results for intraday intra-rater reliability of GM.

Our findings showed excellent ICCs for MT between different investigators, between different measurements of the same investigator as well as between different interpretators. Regardless of image acquisition and interpretation, the variation in assessed MT was very low. Thus, with a minimum of standardization, sonographic MT measurements of GM and VL can be conducted extremely reliable by different investigators and by the same investigator at different measurements as well as evaluated by different interpretators. In contrast, inter-rater and intraday intra-rater reliability testing showed lower ICCs and higher variability of PA and FL for GM and VL. Because ultrasound imaging is usually limited to a two-dimensional view, especially the standardization of the plane of the visualization (probe rotation and orientation) of the three-dimensional muscle structure is crucial. By varying probe rotation and probe orientation just very slightly (e.g., misalignment of perpendicular probe orientation), PA and FL can be under- or overestimated (31). For instance, Klimstra et al. (32) highlighted that changes in probe rotation and orientation can result in a 12% difference in the reported PA. Therefore, variations in PA and FL might be due to subtle differences in probe orientation between different investigators, but also between the measurements of the same investigators. Furthermore, also inter-rater interpretation error was higher for PA and FL for GM and VL compared to MT. Superior and inferior PA as well as FL had to be manually detected on ultrasound images frame by frame. Thus, manual assessment of PA and FL in ultrasound images seems to be very subjective. Additionally, when comparing the reliability of FL between GM and VL, the variations in FL are greater for VL. This might be due to the difference in FL between GM and VL muscles. In fact, the FL of the VL muscle is longer (≈11.1 cm) compared to the GM muscle (≈4.5 cm). The fascicles of the VL muscle extended the field of view during the ultrasound. Therefore, the true length of the fascicle requires estimation instead of directly measuring the FL and therefore is more sensitive to errors. In this context, it is also noticeable that while the variability of FL is greater for VL compared to GM, the ICC values of FL are higher for VL compared to GM. The discrepancy between the ICC and variability may become apparent through the relationship between the accuracy of the ICC and several factors: the sample size, the range of the measuring scale, and the ratio of variances (33). Specifically, the larger the sample size and the wider the measuring scale, the more accurate the ICC. Additionally, higher variability within groups compared to between groups can result in lower ICC values.

In typical sonographic images of healthy skeletal muscle, the hierarchical arrangement is visually represented by parallel striations of hypoechoic muscle fibers and hyperechoic perimysium. SFA is employed to quantify this speckle pattern and the light-dark banding pattern seen in longitudinal B-mode images of healthy muscle, enabling a comprehensive examination and measurement of the characteristic speckle pattern in muscle tissue. While SFA has been predominantly used for investigating tendon tissue, recent research has successfully adapted and extended it for application in skeletal muscle (13, 14). Consequently, SFA may prove to be useful in assessing differences in muscle structure, such as those resulting for instance from training interventions. To utilize SFA for this purpose, it is necessary to verify the reliability of the various SFA parameters to distinguish measurement inaccuracies from training adjustments. Crawford et al. (15) conducted a reliability study investigating hamstring muscles and focusing on four SFA parameters (i.e., PSFR, Mmax, Mmax%, Sum). They found excellent ICCs for the inter-rater interpretation error between different interpreters for the extracted spatial frequency parameters (0.95–0.98) and concluded that SFA may be an objective method for examining changes in muscle tissue due to muscle hypertrophy, swelling, localized edema, or mechanical disruption of the perimysium. Recent updates to the SFA algorithm have introduced other parameters that could be crucial, especially in the context of muscle investigations, for better quantifying aspects like the alignment and packing of muscle fibers (see Table 2). Therefore, the reliability of the new SFA parameters has to be tested, too. Our analyses regarding inter-rater interpretation error indicate excellent ICC values (0.96–0.999) for all SFA parameters, showing that SFA evaluation of the same images with a standardized ROI (performed by different interpreters) is extremely reliable. Nevertheless, in terms of inter-rater and intraday intra-rater reliability, we found moderate to good ICC values with low to high variability. Specifically, Amax and PWP consistently showed the highest ICC values across all analyses but also exhibited the highest variability (ranging from low to high) compared to the low variability of all other tissue organization parameters. Again, this discrepancy between the ICC and variability may become apparent through the previously mentioned relationship between the accuracy of the ICC and several factors (i.e., sample size, range of the measuring scale, ratio of variances). Thus, the SFA parameters appear to respond with different sensitivity to image variations caused by slight changes in the probe position, orientation, and rotation when repositioning the probe. Considering this, the use of special foam casts (17, 20, 34) to standardize probe orientation may be helpful, enabling a constant probe orientation due to rigid probe—skin surface fixation.

A limitation present in this study pertains to the consecutive execution of all measurements for both the test and retest sessions within a single day. This experimental design was intentionally selected to mitigate the influence of confounding variables. Furthermore, it is possible that sitting and subsequently lying down between repeated measurements led to a temporary shift of the skin over the muscle. Insufficient time before the next measurement may prevent the skin from returning to its original position above the muscle, causing the skin marker to not precisely identify the same analysis spot. Nevertheless, we examined the images of several participants for the distance between specific muscular features in the ultrasound image and the skin marker from repeated measurements, and we found no discernible difference in distance. Moreover, the calculation of reliability by averaging data instead of considering each trial separately might be perceived as a form of data smoothing, potentially obscuring to some extent the variability in the data. Additionally, the analyses revealed a disparity between the ICC and the variability (TRV%). As previously mentioned, this incongruity could potentially arise due to the ICC's sensitivity to factors such as sample size, the range of measurement scale, and the variance ratios.

In summary, the present findings have showcased excellent inter-rater and intraday intra-rater reliability concerning MT with minmal variability and systematic bias. However, the agreement was not as robust for the PA, FL, and SFA parameters. Especially Amax and PWP should be interpreted with caution, as they consistently showed high variability (TRV%: <24%). Notably, MT and all SFA parameters exhibited excellent agreement for inter-rater interpretation error. This implies that the updated SFA algorithm holds the promise of objectively and consistently evaluating ultrasound images. To minimize measurement errors, it is advised to standardize probe rotation and orientation. The incorporation of foam casts could potentially facilitate consistent probe orientation by establishing a rigid fixation between the probe and the skin surface. Future research should focus on evaluating the reliability of foam cast scans to ensure the accurate detection of small adaptive changes in muscle architecture and tissue organization.

## Data Availability

The raw data supporting the conclusions of this article will be made available by the authors, without undue reservation.
