# Correction: Reliability of assessing skeletal muscle architecture and tissue organization of the gastrocnemius medialis and vastus lateralis muscle using ultrasound and spatial frequency analysis

**DOI:** 10.3389/fspor.2025.1692168

**Published:** 2025-09-24

**Authors:** Melanie Lesinski, Gregory Bashford, Adrian Markov, Lucie Risch, Michael Cassel

**Affiliations:** ^1^ Division of Training and Movement Sciences, Research Focus Cognition Sciences, University of Potsdam, Potsdam, Germany; ^2^ Department of Biological Systems Engineering, University of Nebraska, Lincoln, NE, United States; ^3^ Department of Sports Medicine, University Outpatient Clinic, University of Potsdam, Potsdam, Germany

**Keywords:** sonography, intraday intra-rater reliability, inter-rater reliability, interpretation error, muscle thickness, pennation angle, fascicle length

There was a mistake in Table 2 as published. An error was made in the description of the physiological correlate for P6. The correct wording is: “a higher P6 value indicates more disorganization of tendon/muscle fibers”. The corrected Table 2 appears below.

**Table 2:** Mathematical description and physiological correlate of spatial frequency parameters.

Parameter	Mathematical description	Mathematical formulation	Physiological correlates
Peak Spatial Frequency Radius (PSFR)	Distance from origin to peak of maximum frequency amplitude in 2-D FFT spectrum	$\sqrt{u_{m}^{2}+v_{m}^{2}}$, where $\left(u_{m},v_{m}\right)$ is the location of the model spectral peak	Most dominant spacing between hyperechoic perimysium of the muscle fascicles and hypoechoic muscle fibers Higher value primarily indicates a tighter packing of tendon/muscle fibers
P6 Width	Euclidian distance of the standard deviation vector of the spatial frequency peak on 2-D FFT spectrum	$\sqrt{\sigma_{u}^{2}+\sigma_{v}^{2}}$, from model fit	A higher P6 value indicates more disorganization of tendon/muscle fibers
Q6 Factor	Ratio of Peak Spatial Frequency Radius to P6 Width	Q6 = $\frac{\mathrm{PSFR}}{P6}$	Normalization factor to faciliate comparision of fiber packing with fiber alignment Higher value primarily indicates a “purer” (parallel) alignment of tendon/muscle fibers.
Amax	Normalized peak value of amplitude spectrum	$\frac{F_{\max}(u,v)}{numel(F)}$	Strength factor of the most dominant spacing of fascicles/fibers Higher value primarily indicates more tendon/muscle fibers per unit volume
PWP	Image power within dominant spacing peak	$\int_{S}\frac{{\left|F(u,v)\right|}^{2}}{numel(F)}$, where *S* is the region of the dominant narrowband peak	Strength factor of fiber spacing close to most dominant spacing Higher value primarily indicates more tendon/muscle fibers per unit volume
PPP	Peak power percent	$\frac{\mathrm{PWP}}{2\int {\left|F(u,v)\right|}^{2}}\times 100\text{\%}$	Strength factor of most dominant spacing of fascicles/fibers as compared to other tissue Higher value primarily indicates more tissue in alignment compared to other tissue in the sample volume

The original version of this article has been updated.

