# Supplementary figures and images for: Reliability of assessing skeletal muscle architecture and tissue organization of the gastrocnemius medialis and vastus lateralis muscle using ultrasound and spatial frequency analysis
Source: Front Sports Act Living. 2024 Jan 18;6:1282031. doi: 10.3389/fspor.2024.1282031 (PMC10830747; doi:10.3389/fspor.2024.1282031)

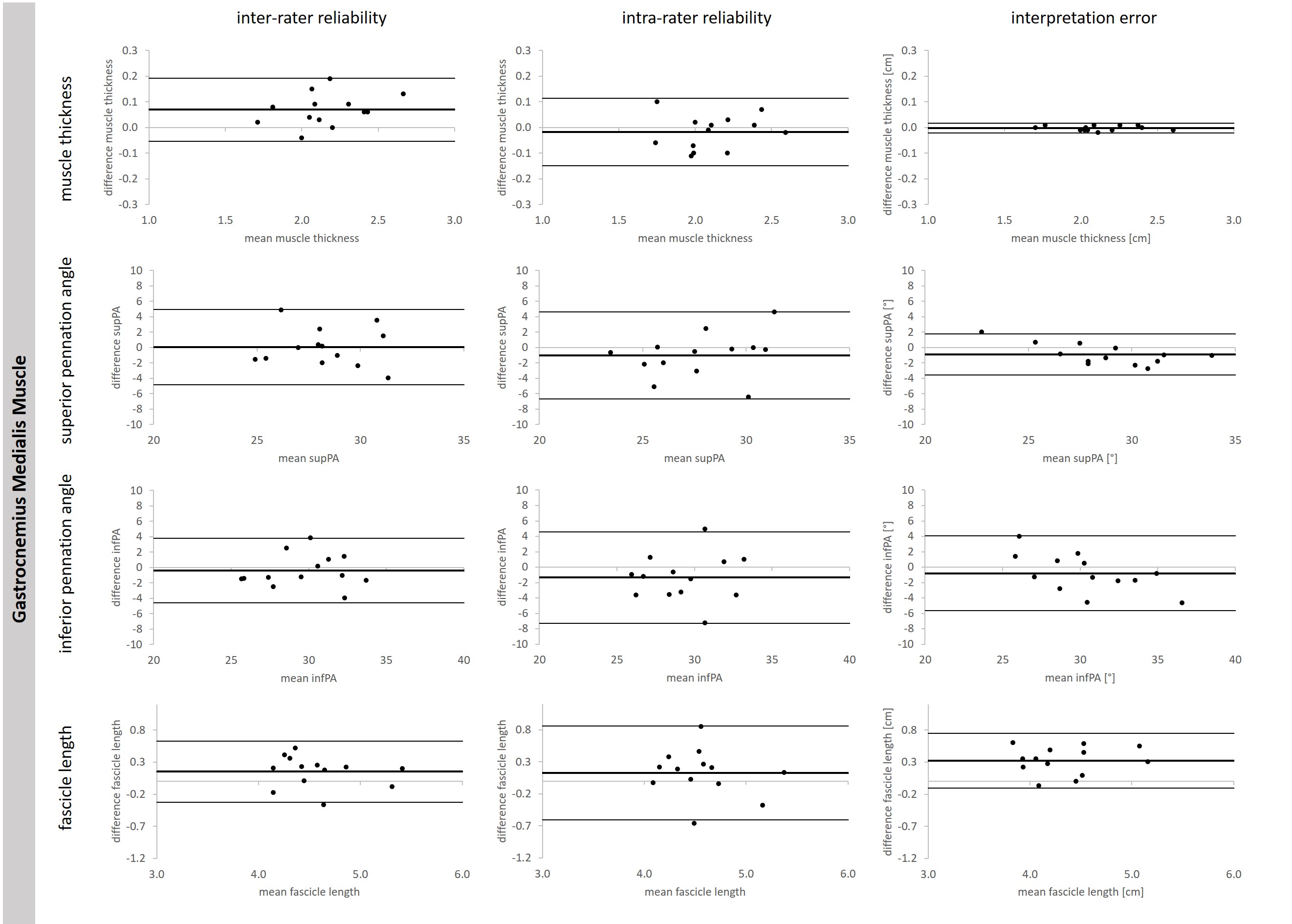

Supplement: Supplementary file 1 [file Image1.jpeg]

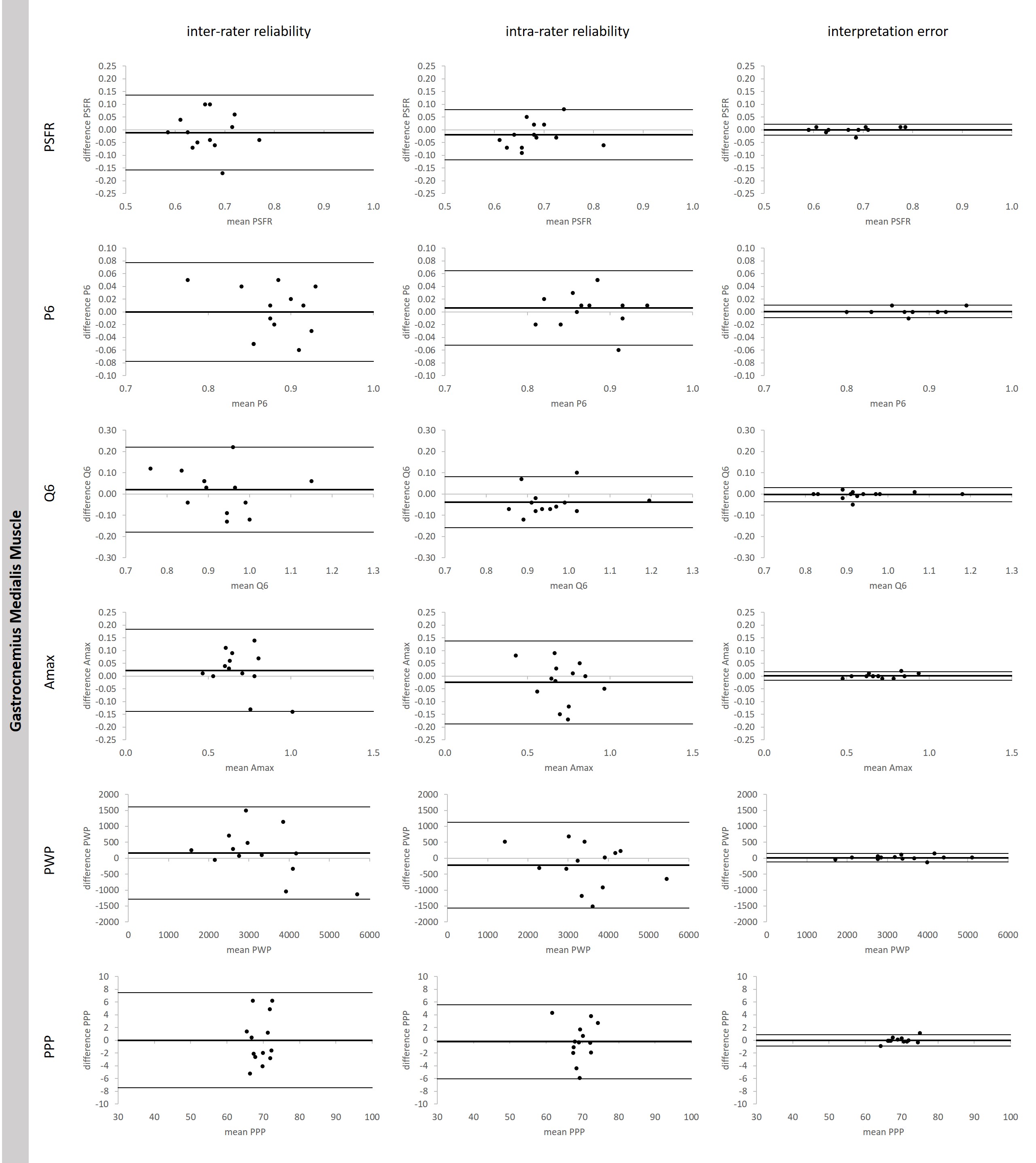

Supplement: Supplementary file 2 [file Image2.jpeg]

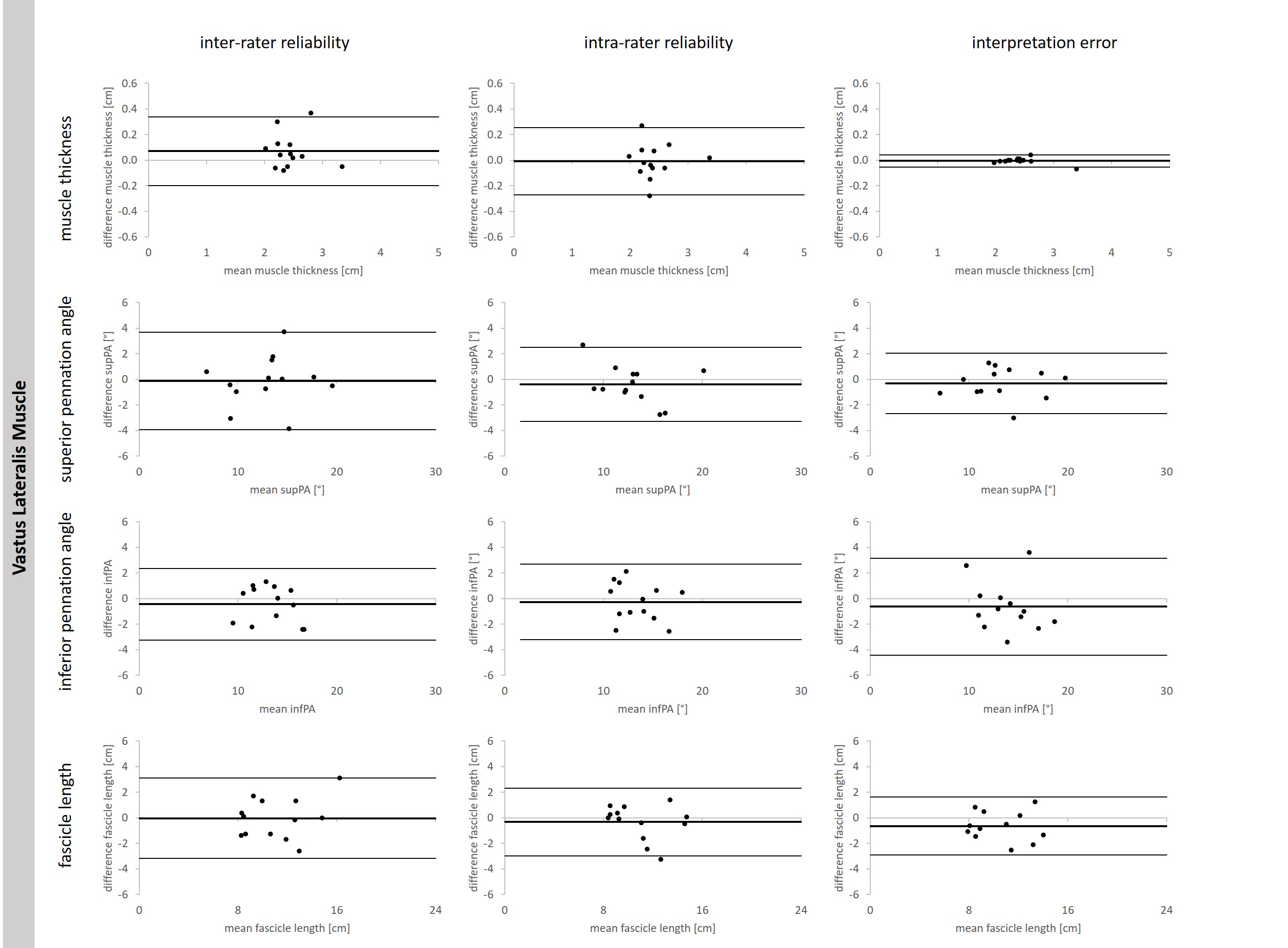

Supplement: Supplementary file 3 [file Image3.jpeg]

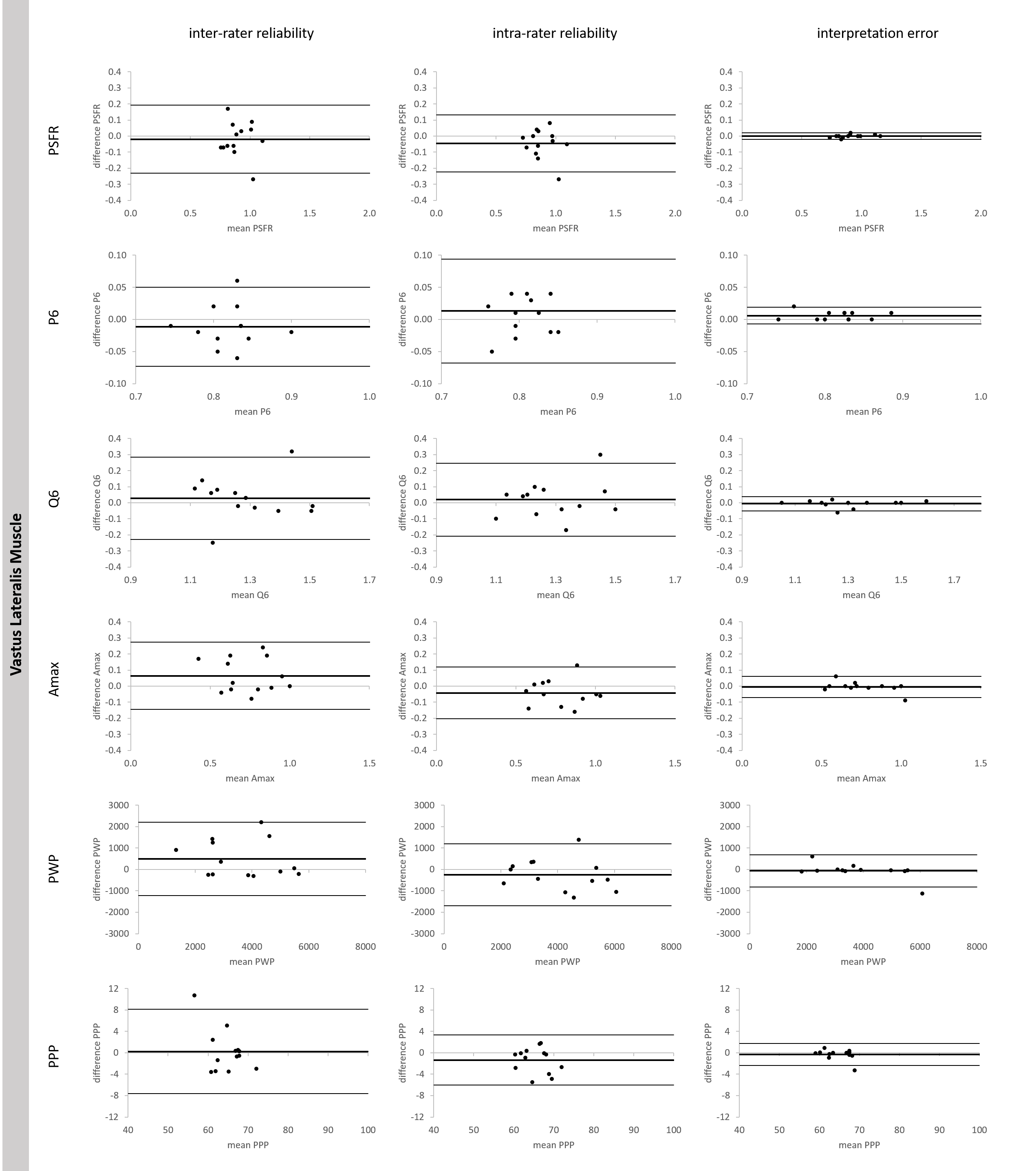

Supplement: Supplementary file 4 [file Image4.png]
